# Asparagine Substitution at PB2 Residue 701 Enhances the Replication, Pathogenicity, and Transmission of the 2009 Pandemic H1N1 Influenza A Virus

**DOI:** 10.1371/journal.pone.0067616

**Published:** 2013-06-14

**Authors:** Bin Zhou, Melissa B. Pearce, Yan Li, Jieru Wang, Robert J. Mason, Terrence M. Tumpey, David E. Wentworth

**Affiliations:** 1 J. Craig Venter Institute, Rockville, Maryland, United States of America; 2 Wadsworth Center, New York State Department of Health, Albany, New York, United States of America; 3 Department of Biomedical Sciences, School of Public Health, State University of New York, Albany, New York, United States of America; 4 Influenza Division, National Center for Immunization and Respiratory Diseases, Centers for Disease Control and Prevention, Atlanta, Georgia, United States of America; 5 Department of Medicine, National Jewish Health, Denver, Colorado, United States of America; Johns Hopkins University - Bloomberg School of Public Health, United States of America

## Abstract

The 2009/2010 pandemic influenza virus (H1N1pdm) contains an avian-lineage PB2 gene that lacks E627K and D701N substitutions important in the pathogenesis and transmission of avian-origin viruses in humans or other mammals. Previous studies have shown that PB2-627K is not necessary because of a compensatory Q591R substitution. The role that PB2-701N plays in the H1N1pdm phenotype is not well understood. Therefore, PB2-D701N was introduced into an H1N1pdm virus (A/New York/1682/2009 (NY1682)) and analyzed *in vitro* and *in vivo*. Mini-genome replication assay, *in vitro* replication characteristics in cell lines, and analysis in the mouse and ferret models demonstrated that PB2-D701N increased virus replication rates and resulted in more severe pathogenicity in mice and more efficient transmission in ferrets. In addition, compared to the NY1682-WT virus, the NY1682-D701N mutant virus induced less IFN-λ and replicated to a higher titer in primary human alveolar epithelial cells. These findings suggest that the acquisition of the PB2-701N substitution by H1N1pdm viruses may result in more severe disease or increase transmission in humans.

## Introduction

Influenza A viruses are important human and animal pathogens that cause frequent epidemics and epizootics, and emergence of novel viruses in humans and animals continue to pose a pandemic threat. Transmission and pathogenesis of influenza A viruses are polygenic traits influenced by the interaction of viral components with host cells. The three RNA polymerase subunits (PB1, PB2, and PA) are encoded by different genomic RNA segments and the proteins interact to form a heterotrimeric RNA dependent RNA polymerase (RdRp), which transcribes and replicates the eight different nucleoprotein (NP) encapsidated genomic RNA segments that comprise the viral genome. The RdRp and the NP are major determinants of host species-specificity, transmission, and pathogenesis. The 2009 influenza A pandemic was caused by H1N1 subtype viruses (H1N1pdm), which have a triple reassortant RNA polymerase and NP gene constellation that contains avian (PB2, and PA), human (PB1) and swine (NP) lineage gene segments [1–3]. It is anticipated that additional adaptations will evolve in the H1N1pdm viruses unique triple reassortant gene constellation as it adapts to humans, and these changes have the potential to enhance its virulence and/or transmission.

The avian-lineage PB2 gene of H1N1pdm is one of the gene segments likely to undergo evolutionary selection in the new human host. This avian-lineage PB2 lacks some characteristic human/mammalian amino acid signatures, such as lysine at residue 627 (PB2-627K) or asparagine at residue 701 (PB2-701N). Several groups have shown that introduction of the PB2-E627K substitution into H1N1pdm viruses had little effect on the pathogenesis and/or transmission of H1N1pdm viruses in mice and ferrets [4–8]. This was unexpected to some extent, since many studies have shown that introduction of PB2-627K into H5N1 and other zoonotic avian influenza A virus subtypes increased virulence and transmissibility in mammalian models [9–13]. However, this contradiction was nicely explained by two studies showing that a unique substitution (PB2-Q591R) in the avian-lineage PB2 of the H1N1pdm virus compensated for the PB2-627E commonly found in human H1N1pdm viruses [6,14].

PB2-701N has also been shown to compensate for the lack of PB2-627K and enhance the replication of some strains of influenza A viruses in mammalian hosts [15]. For example, studies with the mouse adapted avian H7N7 influenza virus SC35M showed that PB2-701N increased the polymerase activity and enhanced virulence in mice and PB2-701N in the avian H5N1 virus DKGX/35 showed enhanced pathogenesis in mice and that it was a prerequisite for transmission in guinea pigs [16]. Additionally, Steel et al., demonstrated the compensatory effects of PB2-701N on avian and human viruses containing PB2-627E using the guinea pig transmission model [12]. We hypothesized that since the PB2-D701N substitution has been observed in some zoonotic avian H5N1 viruses and some avian-lineage swine influenza viruses [15–17], it may provide an advantage to H1N1pdm viruses as their avian-lineage PB2 gene segment evolves in the human population. Therefore, to evaluate the potential role that PB2-D701N may play in the adaptation of H1N1pdm viruses to humans, we conducted a comprehensive study to determine the influence that PB2-D701N has on viral replication and transmissibility. The results show that although the wild type H1N1pdm virus contained the PB2-591R polymorphism, the PB2-D701N substitution increased the viral polymerase activity in human cell lines, conferred more efficient virus replication in a restrictive mouse epithelial cell line, increased virulence in the BALB/c mouse model, and increased virus transmission in the ferret transmission model. Additionally, the PB2-D701N virus reduced secretion of the antiviral IFN-λ cytokines and showed enhanced replication in primary human alveolar epithelial cells relative to the wild type H1N1pdm virus.

## Materials and Methods

### Biosafety and ethics statement

This study was reviewed and approved by the Wadsworth Center Institutional Biosafety Committee, the National Jewish Institutional Biosafety Committee, and it was reviewed under the CDC procedures for identification of dual use research of concern. All experiments with infectious virus were performed using procedures and facilities that met or exceeded the requirements set forth by the U.S. Department of Health and Human Services for propagation of influenza A viruses. *In vitro* experiments with infectious H1N1pdm viruses were conducted in biosafety level 2 laboratory as described by the Centers for Disease Control and Prevention interim biosafety guidelines. *In vivo* experiments were carried out in strict accordance with the recommendations in the Guide for the Care and Use of Laboratory Animals of the National Institutes of Health. Mouse study was performed in a biosafety level 3 laboratory approved for such use by the Centers for Disease Control and Prevention and the U.S. Department of Agriculture and were conducted under animal care and use protocols approved by Wadsworth Center’s Institutional Animal Care and Use Committee (IACUC protocol 09-377). No survival surgery was performed, and all efforts were made to minimize suffering. All ferret procedures were approved by Institutional Animal Care and Use Committee (IACUC) of the Centers for Disease Control and Prevention and in an Association for Assessment and Accreditation of Laboratory Animal Care International-accredited facility. Animal studies were performed in accordance with the IACUC guidelines under protocol #2195TUMFERC-A3: “Studies on the Pathogenesis and Transmission of Recombinant Influenza Viruses in Ferrets”.

### Cell lines

Human embryonic kidney 293T (HEK-293T) cells and mouse rectum epithelial carcinoma (CMT-93) cells were maintained in Dulbecco’s modified Eagle’s medium (DMEM) supplemented with 10% fetal bovine serum (FBS). Madin-Darby canine kidney (MDCK) cells were maintained in Eagle’s minimum essential medium (EMEM) supplemented with 5% FBS. Human lung epithelial (Calu-3) cells were maintained in EMEM supplemented with 10% FBS, 1% nonessential amino acids, and 1 mM sodium pyruvate. MDCK, CMT-93, and Calu-3 cells were obtained from the American Type Culture Collection, Rockville, MD.

### Generation of recombinant viruses

Recombination-based In-Fusion cloning method was used to mutate the NY1682 PB2 plasmid to generate the PB2-D701N mutant reverse genetics construct [18–20]. The recombinant rNY1682-WT and rNY1682-D701N viruses were generated using the rescue protocol described previously [21]. Briefly, 0.6 µg of plasmid for each gene segment was mixed and incubated with 15 µl of Lipofectamine 2000 (Invitrogen, Carlsbad, CA) at 20°C for 20 min. The Lipofectamine-DNA mixture was transferred to 90% confluent 293T/MDCK cell monolayers in a 35-mm tissue culture dish and incubated at 33°C with 5% CO_2_ for 8 h. The transfection supernatant was replaced with 3 ml of Opti-Mem I medium (Invitrogen) supplemented with 0.3% bovine serum albumin (BSA) fraction V (Invitrogen), 3 µg/ml tosylsulfonyl phenylalanyl chloromethyl ketone (TPCK)-trypsin (Worthington, Lakewood, NJ), and 1% antibiotic-antimycotic (Invitrogen). Three days post-transfection, the supernatant was collected and viruses were propagated in MDCK cells at 33°C. Titers of the viruses used in this study were determined by TCID_50_ and/or plaque assay in MDCK cells.

### Mini-genome replication assay

The luciferase-mediated mini-genome replication assay was performed as previously described, using a PolI-driven reporter plasmid and pDZ-based PB2, PB1, PA, and NP bidirectional expression plasmids [18,22]. Briefly, HEK-293T cells in 24-well plates were cotransfected with 0.2 µg each of pPolI-NS-Luc plasmid (pBZ81A36) and plasmids to express the NY1682 PB2 (PB2-WT or PB2-D701N), PB1, PA, and NP proteins, and to control for transfection efficiency, 0.02 µg of the Renilla luciferase plasmid pRL-TK (Promega, Madison, WI) was also cotransfected. Cells were incubated at 33^o^C, 37^o^C, and 39^o^C for 6 or 18 hours, and then luciferase production was assayed using the dual-luciferase reporter assay system (Promega) according to the manufacturer’s instructions. Firefly luciferase expression was normalized to Renilla luciferase expression (relative activity). At each temperature, the relative activity of WT polymerase was set at 100%, and the activities of the PB2-D701N were determined relative to that of the WT. All results shown are the averages from triplicate experiments, and the standard deviation is shown.

### Replication kinetics *in vitro*

MDCK or CMT-93 cell monolayers in 12-well plates were washed twice with PBS, and then 2 ml of virus growth medium (VGM) was added to each well. The cells were inoculated at a multiplicity of infection (MOI) of 0.01 TCID_50_/cell with recombinant NY1682 WT or NY1682 D701N viruses. Supernatants were collected at 2, 24, 48, 72 (and 96) hours post inoculation (hpi). Infections of Calu-3 cells were performed similarly, except that an MOI of 0.02 TCID_50_/cell was used and cultures were maintained at three different temperatures (33^o^C, 37^o^C, and 39^o^C) after inoculation. The VGM used for MDCK and CMT-93 cells was EMEM supplemented with 0.15% BSA fraction V, 2 µg/ml TPCK-trypsin, and 1% antibiotic-antimycotic, and the VGM used for Calu-3 cells was EMEM supplemented with 0.3% BSA fraction V, 1 µg/ml TPCK-trypsin, and 1% antibiotic-antimycotic. All virus titers were determined by TCID_50_ assay using MDCK cells.

### Replication and cytokines secretion in primary human alveolar epithelial cells

Alveolar type II (ATII) cells were isolated from de-identified human lungs that were not suitable for transplantation but donated for medical research as described previously [23]. The Committee for the Protection of Human Subjects at National Jewish Medical and Research Center approved this research. To transdifferentiate type II cells into type I-like cells, type II cells were plated on tissue culture plastic plates at a density of 2.0X10^5^/cm^2^ in DMEM with 10% FBS [24]. After 24-48 h of adherence, the medium was changed to DMEM with 5% FBS and cells were cultured for 6 days and then used for virus infection.

The primary cultured alveolar type I-like cells were infected with the rNY1682-WT or rNY1682-D701N viruses at an MOI of 0.01 PFU/cell and washed twice with medium after 1 h of incubation at 37^o^C. Culture supernatants of the infected cells were collected at 1, 12, 24, 48, and 72 hpi and viral titers were determined using plaque assay in MDCK cells. Supernatants collected at 24 hpi were used to determine the protein levels of selected cytokines/chemokines using specific enzyme-linked immunesorbent assays (ELISAs) as described previously [25]. The ELISA kits for human IFN-λ, CCL5, IL-6, and IL-8 were purchased from ELISA Tech (ELISA Tech, Aurora, CO).

### Mouse experiments

To determine virus replication *in vivo*, six-week-old female BALB/cJ mice (n=3/group/time-point, Jackson Laboratory, Bar Harbor, ME) were anesthetized with isoflurane and inoculated intranasally with 50 µl EMEM diluent containing 10^3^ TCID_50_ of rNY1682-WT or rNY1682-D701N. Animals were euthanized at 12, 24, 48, 96 hpi. The entire lung of each animal was homogenized in 1 ml of media and clarified by centrifugation, and nasal washes were collected from each mouse in 1 ml of media. Virus titers in the lungs and nasal washes were determined by TCID_50_ assay.

To determine morbidity and mortality, groups of five-to-six-week-old female BALB/cJ mice (n=5/group) were inoculated intranasally with the recombinant viruses at the indicated doses in 50 µl of EMEM diluent or mock inoculated with EMEM to serve as controls. Body weight was measured daily for 14 days, and clinical observations were recorded. Mice that lost more than 25% of their original weight were euthanized for humane reasons using Avertin anesthesia followed by bilateral thoracotomy or cervical dislocation.

### Ferret transmission experiments

Male Fitch ferrets (Triple F Farms, Sayre, PA), six months of age and serologically negative by hemagglutination inhibition (HI) assay for currently circulating influenza viruses, were used in this study. In a Duo-Flow Bioclean environmental enclosure (Lab Products, Inc., Seaford, DE), ferrets were housed in adjacent transmission cages, each modified so that a side wall was replaced with a stainless-steel, perforated wall with holes 1–5 mm in diameter and spaced 3 mm apart to facilitate the transfer of respiratory droplets through the air while preventing direct contact between ferrets and indirect contact with the bedding and food of neighboring ferrets [26,27]. A total of six ferrets were used for each respiratory droplet transmission experiment. Three ferrets were inoculated intranasally with 1 ml of 10^6^ PFU of the NY1682-WT or NY1682-D701N virus. Twenty-four hours after inoculation, three naive ferrets were each placed in a cage adjacent to an inoculated ferret. Ferrets were monitored for clinical signs through 14–18 dpi as previously described. Nasal washes were collected on 1, 3, 5, 7, and 9 dpi from the inoculated ferrets and were collected on 1, 3, 5, 7, 9, and 11 dpc (days post-contact) from the contact ferrets. Viral titers were determined in MDCK cells by plaque assay. HI analysis was also performed on post-exposure ferret sera collected 14–18 dpc using 0.5% turkey erythrocytes against homologous virus.

### Statistics

Statistical analyses were conducted in GraphPad Prism version 5.0 (GraphPad Software, San Diego, CA) using ANOVA analysis with Bonferroni post-test. For simple comparisons, Student’s *t* test with two-tailed analysis was used as indicated in the text.

## Results

### PB2-D701N substitution increases viral RNA polymerase activity *in vitro*

**Figure 1:**
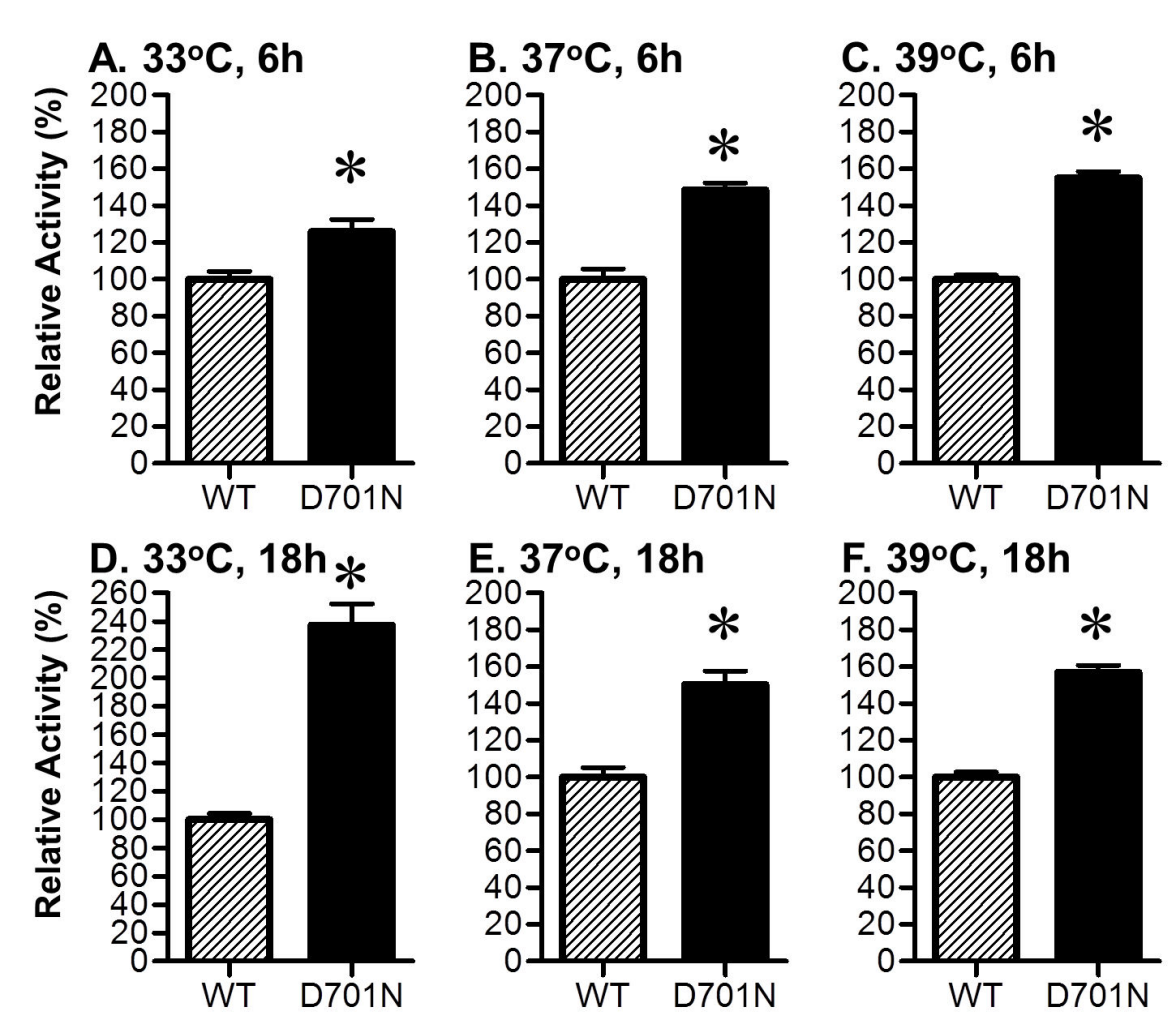
PB2-D701N substitution enhances viral RNA polymerase activity in human cells. HEK-293T cells were transfected with pPolI-NS-Luc plasmid (pBZ81A36) that expresses negative sense virus-like RNA encoding a destabilized firefly luciferase enzyme that can be transcribed by the viral RdRp. The HEK-293T cells were also co-transfected with plasmids expressing the NY1682 PB1, PA and NP, and one of the PB2 clones (WT, or D701N) to generate different viral RdRp. Cells were also co-transfected with a Renilla luciferase expression plasmid to control for transfection efficiency. After 6 h and 18 h incubation at 33^o^C, 37^o^C, or 39^o^C, both firefly and Renilla luciferase production were measured, and Renilla expression was used to normalize the data. The averages of triplicate experiments are shown with error bars that represent standard deviation (SD). * Indicates that the difference between the PB2-WT and PB2-D701N was statistically significant (p<0.05, t-test).

We used a luciferase-mediated mini-genome assay to examine the effects of the PB2-D701N substitution on the H1N1pdm viral polymerase activity. Human embryonic kidney-293T cells (HEK-293T) were co-transfected with plasmids expressing the NY1682 PB2 (either WT or D701N), PB1, PA, NP proteins, and a pPolI-NS-Luc reporter plasmid [8]. After 6 h or 18 h incubation at 33^o^C, 37^o^C, or 39^o^C, luciferase production was assayed. At both time points and all temperatures, the PB2-D701N substitution resulted in a significantly higher level of relative luciferase activity compared to the PB2-WT (p<0.05, t-test) (Figure 1), indicating that the D701N substitution in the PB2 protein enhances activity of the H1N1pdm RdRp. The PB2-D701N substitution also increased the H1N1pdm polymerase activity in A549 cells, a human lung epithelial cell line (data not shown). Using the same assay, we have previously shown that introduction of E627K into NY1682-PB2 increased polymerase activity by 400% and E158G substitution in NY1682-PB2 increased activity by 1500% [8]. Although the increased RNA polymerase activity conferred by PB2-D701N was fairly modest in comparison to other mutations (e.g., E627K, and E158G) [8], seemingly small differences in RNA polymerase activity may still influence transmission or pathogenesis. Therefore, the effects of the PB2-D701N substitution on virus replication, pathogenesis, and transmission were investigated.

### The rNY1682-D701N virus replicates more efficiently in mouse epithelial cells and in human lung epithelial cells at an elevated temperature

**Figure 2:**
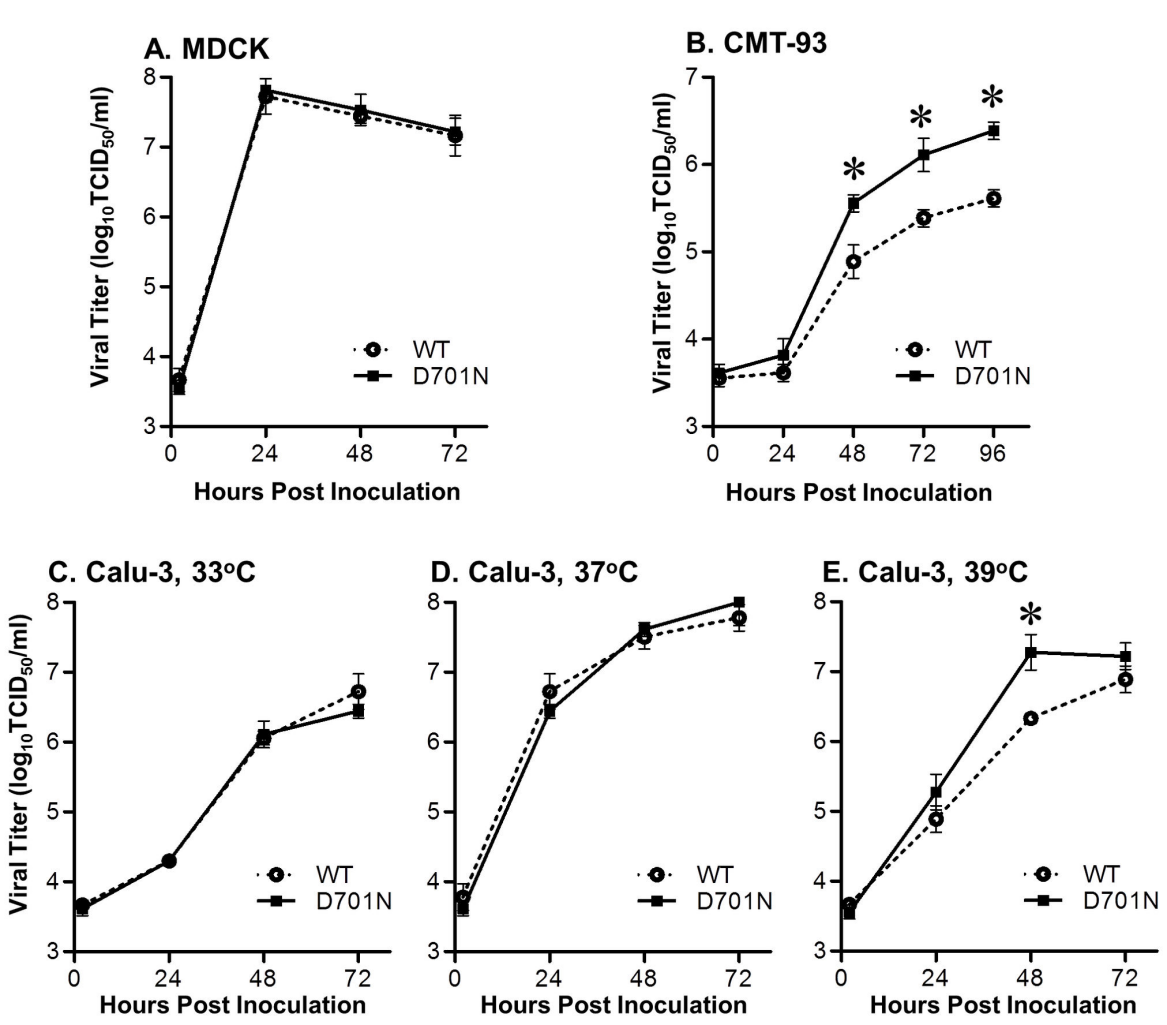
Replication kinetics of rNY1682-WT and rNY1682-D701N *in* *vitro*. (**A**) Confluent monolayers of Madin-Darby canine kidney (MDCK) cells were inoculated with the rNY1682-WT (**WT**) or rNY1682-D701N (**D701N**) viruses at 0.01 TCID_50_/cell. Supernatants were collected at 2, 24, 48 and 72 hours post inoculation (hpi). (**B**) Confluent mouse rectal epithelial carcinoma cells (CMT-93) were inoculated with rNY1682-WT (**WT**) or rNY1682-D701N (**D701N**) viruses at 0.01 TCID_50_/cell and supernatants were harvested at 12, 24, 48, 72 and 96 hpi. (**C**, **D**, **E**) Confluent human lung epithelial cells (Calu-3) were inoculated with rNY1682-WT (**WT**) or rNY1682-D701N (**D701N**) viruses at 0.02 TCID_50_/cell and supernatants were harvested at 2, 24, 48, 72 and 96 hpi. Cells were incubated at 33^o^C (**C**), 37^o^C (**D**), or 39^o^C (**E**) for the respective growth curves. The averages of triplicate experiments are shown with error bars (+/-) representing the standard deviation, and * indicates that the differences were statistically significant (p<0.001, ANOVA).

Previous studies have shown that D701N substitution in the PB2 protein of H5N1 virus can improve virus growth in mammalian cells [17,28]. To determine if the D701N substitution also has a similar effect on the H1N1pdm virus, we created a recombinant H1N1pdm virus that differed from the wild-type A/New York/1682/2009 (rNY1682-WT) at PB2-D701N (rNY1682-701N). The growth kinetics of the rNY1682-WT and rNY1682-D701N viruses was almost identical in MDCK cells, and both viruses reached peak titers of 10^7.7^ TCID_50_/ml by 24 hpi (Figure 2A). These data are consistent with results obtained by Herfst et al.*,* in the background of another H1N1pdm strain, rA/Netherlands/602/2009 virus [7].

Since MDCK cells are an ideal substrate for the replication of influenza viruses and even attenuated viruses often replicate efficiently in this cell line, we also compared the replication kinetics of these two viruses in a restrictive mouse epithelial cell line (CMT-93) that we had previously found to be more sensitive to the effects of RdRp mutations than MDCK and some other cell lines [8]. In the CMT-93 cells, the rNY1682-D701N replicated more efficiently than rNY1682-WT, and the titers of rNY1682-D701N were significantly higher (~8-fold) than that of rNY1682-WT at 48, 72 and 96 hpi (p<0.001, ANOVA) (Figure 2B).

Although the mini-genome assay results indicate that the D701N substitution increases the polymerase activity in human kidney and lung cell lines (HEK-293T and A549), rNY1682-WT and rNY1682-D701N showed similar growth kinetics in the human lung Calu-3 cell line at 33^o^C and 37^o^C (Figure 2C and D). Interestingly, at 39^o^C, rNY1682-D701N replicated faster and the viral titer was modestly higher than rNY1682-WT at 48 hpi (p<0.001, ANOVA) (Figure 2E). The higher temperature may modestly restrict replication of the rNY1682-WT virus replication in Calu-3 cells.

### PB2-D701N reduces induction of IFN-λ and enhances virus replication in primary human alveolar epithelial cells

**Figure 3:**
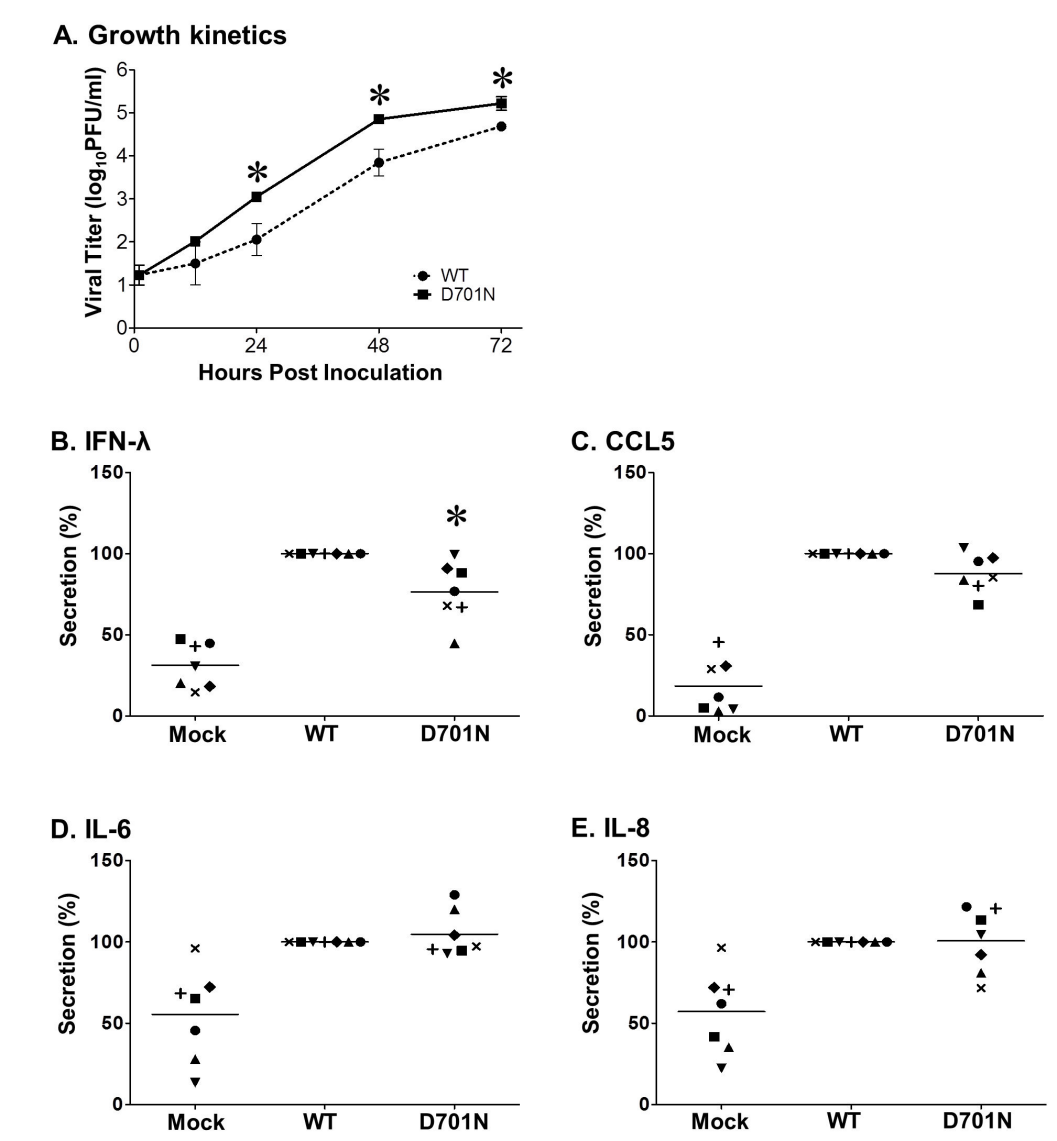
Analysis of cytokine response and virus replication in primary human alveolar epithelial cells. Human alveolar type I-like cells were infected at 37°C with MOI of 0.01 PFU/cell of the rNY1682-WT (**WT**) or rNY1682-D701N (**D701N**) virus, or were mock infected (**Mock**). Supernatants from the culture were collected at various time points post infection. (**A**) Supernatants collected at 1, 12, 24, 48, and 72 hpi were determined for viral titers by plaque assay. Representative growth kinetics of the **WT** and **D701N** viruses in one donor cells are shown. Supernatants collected at 24 hpi were analyzed by ELISA for the levels of (**B**) IFN-λ, (**C**) CCL5, (**D**) IL-6, and (**E**) IL-8. Each symbol represents an average value of 2 or 3 repeats using the same cells from one donor and thus seven different symbols represent the data from seven independent experiments performed using cells from seven donors. Due to the variation in cytokine responses among different donors, for each donor, cytokine/chemokine induced by **WT** was set as 100% and cytokine/chemokine induced by **D701N** or **Mock** was presented as a percentage of **WT**. Horizontal bar in each group indicates the average value from seven donors and * indicates a statistical difference between WT and D701N (p<0.05, ANOVA).

The increased virus replication in restricted cell lines prompted us to examine the viruses in primary human alveolar epithelial cells. About 95% of the alveolar epithelium is covered by flattened alveolar type I (ATI) cells [29], which makes them an important target for respiratory viruses in the gas exchange portions of the lung. Although isolation and culture of human ATI cells have not been reported to our knowledge, Wang et al.*,* transdifferentiated alveolar type II cells into type I-like cells and established the virus infection model in these cells [30–32]. To determine if the PB2-D701N substitution influences the replication of the H1N1pdm virus in human lung cells, primary cultured ATI-like cells were prepared from lungs of seven de-identified donors as described previously [23], and were infected with the rNY1682-WT and rNY1682-D701N viruses at 37^o^C. The culture supernatants were collected at different time points post infection (1, 12, 24, 48, and 72 hpi) and the virus titers were determined by plaque assay. Although viral titer from different donors varied, we found that the rNY1682-D701N virus always replicated to modestly higher titers than the rNY1682-WT virus (Figure 3A). Therefore, the ATI-like cells may be more sensitive than the Calu-3 cells for the detection of the effects from the PB2-D701N mutation (compare to Figure 2D). It is possible that at the optimal temperature (37°C), the rNY1682-WT virus replicated to very high titers (10^7.8^ TCID_50_/ML) in the Calu-3 cells (Figure 2D), to which the rNY1682-D701N virus was unable to surpass. In contrast, the rNY1682-D701N replicated faster and to higher titer than rNY1682-WT virus in ATI-like cells. Taken together, the rNY1682-D701N virus consistently demonstrated similar or enhanced replication kinetics compared to the rNY1682-WT (Figures 2 and 3).

The growth advantage of the rNY1682-D701N virus in ATI-like cells could be the result of interaction between the viral factor, PB2-D701N, and multiple host factors. To examine host factors involved in the different virus growth kinetics, we focused on selected cytokines and chemokines that are important for host immune response to influenza viruses. At 24 hpi, supernatants from infected cells were collected and examined by ELISA for the levels of IFN-λ, CCL5, IL-6, and IL-8. Compared to the rNY1682-WT infected cells, rNY1682-D701N infected cells secreted significantly lower amount of IFN-λ (Figure 3B), which represents important virus induced antiviral type III interferon (IL28/29). In contrast, rNY1682-WT and rNY1682-D701N infected cells secreted comparable levels of CCL5 (Figure 3C), IL-6 (Figure 3D), and IL-8 (Figure 3E). Additionally, we performed a similar experiment (one donor) using an MOI of 3 and detected the same trend in IFN-λ response (rNY1682-D701N lower than rNY1682-WT, data not shown). The lower level of IFN-λ Induced by the rNY1682-D701N virus may give the virus a replication advantage in the type I-like cells.

### PB2-D701N enhances lung replication and pathogenicity of the H1N1pdm virus in a mouse model

**Figure 4:**
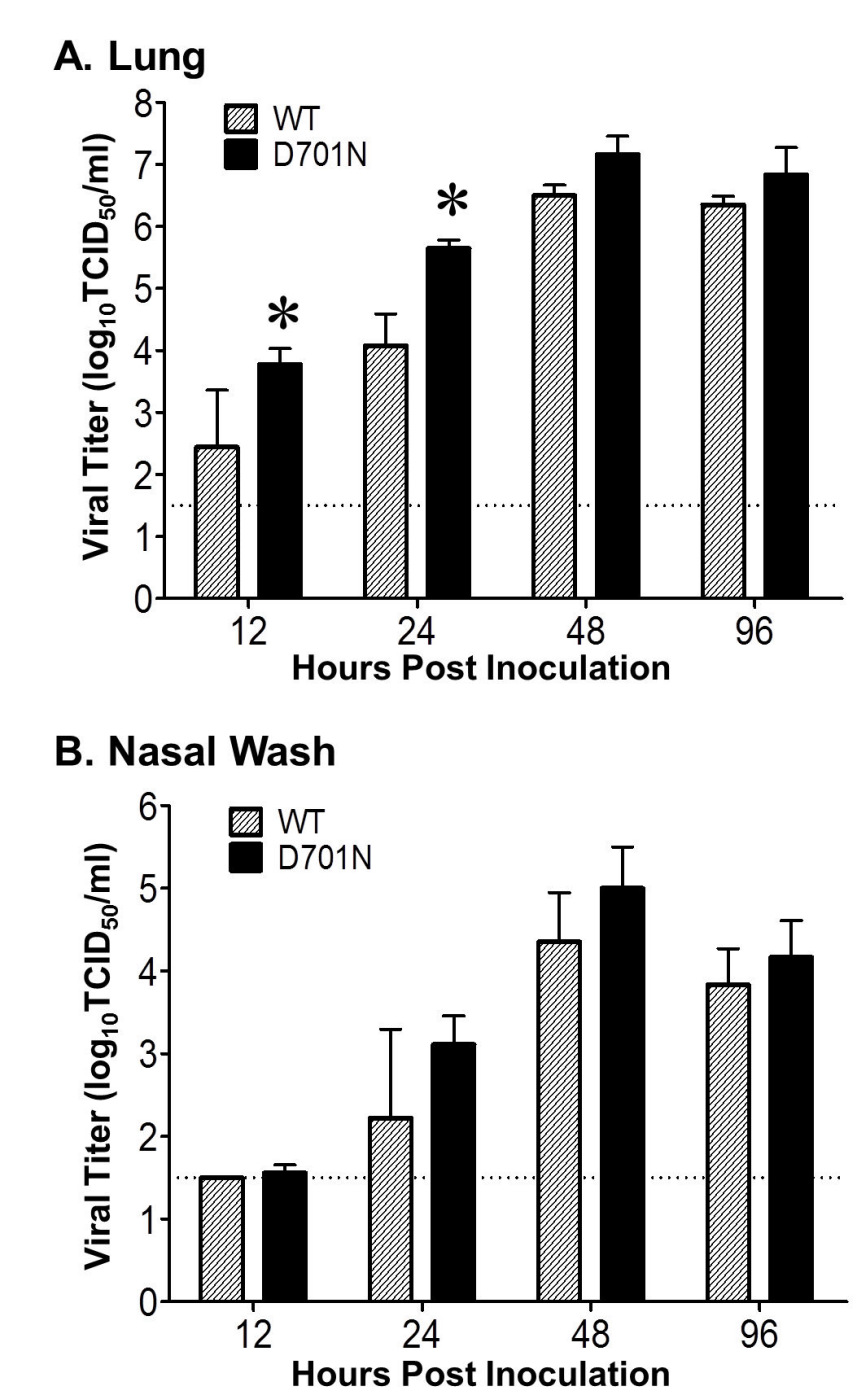
Analysis of viral replication efficiency in the respiratory tracts of mice. Six-week-old female BALB/cJ mice (n=3/group/time-point) were inoculated intranasally with 50 µl containing 10^3^ TCID_50_ of rNY1682-WT (**WT**) or rNY1682-D701N (**D701N**). Animals were euthanized at 12, 24, 48, 96 hpi. The entire lung of each animal was homogenized in 1 ml of media and clarified by centrifugation, and nasal washes were collected from each mouse in 1 ml of media. (**A**) Viral titers of clarified lung homogenates or (**B**) nasal washes were determined by TCID_50_ assay using MDCK cells. The average of each group is shown with error bars representing SD(+/-), and * indicates a statistical difference between WT and D701N (p<0.05, ANOVA). The dotted lines at 1.5 log_10_ indicate the lower limit of detection of infectious virus.

To determine if the PB2-D701N substitution also enhances H1N1pdm virus replication in vivo, six-week-old female BALB/cJ mice were anesthetized with isoflurane and inoculated intranasally with 50 µl virus diluent containing 10^3^ TCID_50_ of either rNY1682-WT or rNY1682-D701N. Compared to rNY1682-WT, rNY1682-D701N replicated more rapidly and had 40-fold higher titers in the lungs as early as 24 hpi (Figure 4A). Statistical analysis of the data demonstrated that the rNY1682-D701N infected mice had significantly higher titers at 12 and 24 hpi (p<0.05, ANOVA), whereas the modest titer differences in the nasal washes at 24 and 48 hpi (Figure 4B) were not statistically significant compared to the rNY1682-WT (p>0.05, ANOVA). The dramatic increase in viral titer in the lungs of mice infected by rNY1682-D701N indicate that this substitution has a stronger impact on replication *in vivo* than in the mouse CMT-93 cell line, which didn’t show a difference between rNY1682-WT and rNY1682-D701N until 48 hpi (Figure 2B).

**Figure 5:**
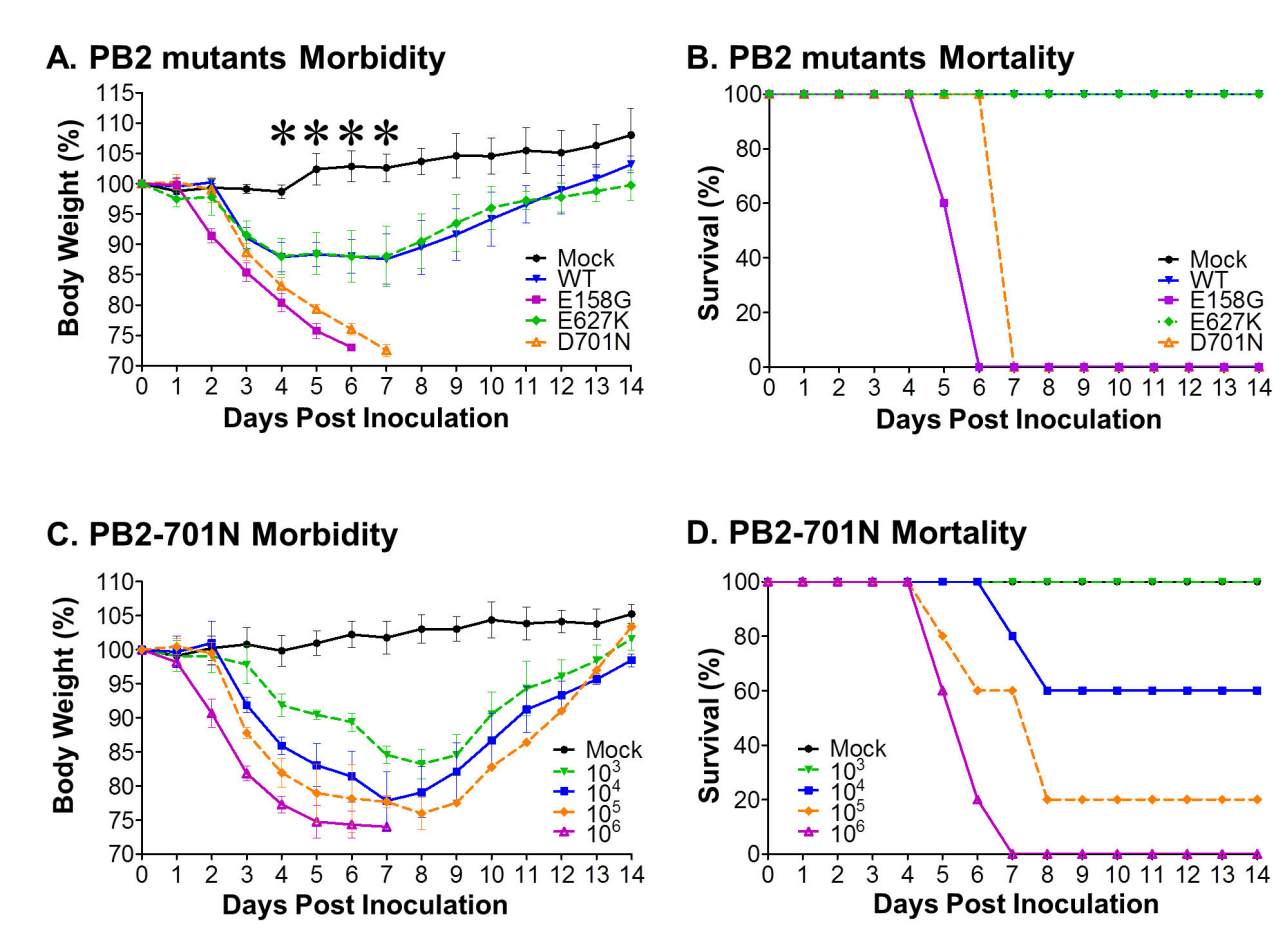
Pathogenicity of rNY1682-WT, rNY1682-E158G, rNY1682-E627K, and rNY1682-D701N viruses in mice. Six-week-old female BALB/cJ mice (n=5/group) were inoculated intranasally with 50 µl containing 10^5^ TCID_50_ of different recombinant viruses (rNY1682-WT (**WT**), rNY1682-E158G (**E158G**), rNY1682-E627K (**E627K**), or rNY1682-D701N (**D701N**)) or they were mock inoculated (**Mock**). (**A**) Morbidity was assessed by weight loss over a 14 day period and is graphed as a percentage of the animals weights on the day of inoculation (day 0). The average body weight of each group is shown with error bars representing SD (+/-), and a significant difference between D701N and WT or E627K was evident as early as 4 d.p.i (*, p<0.01, ANOVA). (**B**) Mortality associated with infection by the recombinant viruses (rNY1682-WT (**WT**) or rNY1682-D701N (**D701N**)) was also examined. Five-week-old female BALB/cJ mice (n=5/group) were inoculated intranasally with 50 µl containing 1×10^3^, 10^4^, 10^5^, or 10^6^ TCID_50_ of rNY1682-D701N or they were mock inoculated. (**C**) Morbidity was assessed by weight changes over a 14 day period and it is graphed as a percentage of the weight on the day of inoculation (day 0). The average body weight of each group is shown with error bars representing SD. (**D**) Mortality of the mice was also monitored for 14 days post inoculation. In accordance with our animal welfare protocol, mice that lost more than 25% of their original weight were euthanized for humane reasons.

The rapid and robust replication of rNY1682-D701N in mouse respiratory tract warranted further evaluation of the effect of PB2-D701N on the pathogenicity of H1N1pdm virus. We compared H1N1pdm viruses that expressed wild type PB2 (rNY1682-WT), PB2-E627K (rNY1682-E627K), PB2-D701N (rNY1682-D701N), or a PB2-E158G (rNY1682-E158G) substitution that we previously showed significantly increases the pathogenicity of the H1N1pdm virus [8]. BALB/cJ mice were inoculated intranasally with 10^5^ TCID_50_ of each virus and monitored for clinical signs and weight loss for 14 days (n=5/group). Mice inoculated with rNY1682-WT, rNY1682-E627K and rNY1682-D701N, began to lose weight by 3 dpi; and more significant weight loss was observed in the mice inoculated with rNY1682-D701N than in mice inoculated with rNY1682-WT or rNY1682-E627K as early as 4 dpi (p<0.01, ANOVA) (Figure 4A). The mice inoculated with rNY1682-WT or rNY1682-E627K had an average maximum weight loss of approximately 12% and began to recover by 8 dpi (Figure 5A). In contrast, the group of mice infected by rNY1682-D701N continued to lose weight throughout the experiment and all of these animals succumbed to infection by 7 dpi (Figure 5A and 5B).

To further evaluate the influence of PB2-D701N, we determined the 50% mouse lethal dose (MLD50) by intranasally inoculating BALB/cJ mice with 10^3^, 10^4^, 10^5^, or 10^6^ TCID_50_ of rNY1682-D701N; all of the groups of mice showed prominent weight loss (Figure 5C), and the mortality rate was 0%, 40%, 80%, and 100%, respectively (Figure 5D). The MLD50 of rNY1682-D701N was 2X10^4^ TCID_50_, which is at least 300-fold lower than that of the parental virus rNY1682-WT (>6X10^6^ TCID_50_) and 33-fold higher than that of the rNY1682-E158G virus (6X10^2^ TCID_50_) [8]. These data show that rNY1682-D701N is more virulent than either rNY1682-WT or rNY1682-E627K, and that it is less virulent than the highly pathogenic rNY1682-E158G virus.

### PB2-D701N increases the transmissibility of the H1N1pdm virus in a ferret model

**Figure 6:**
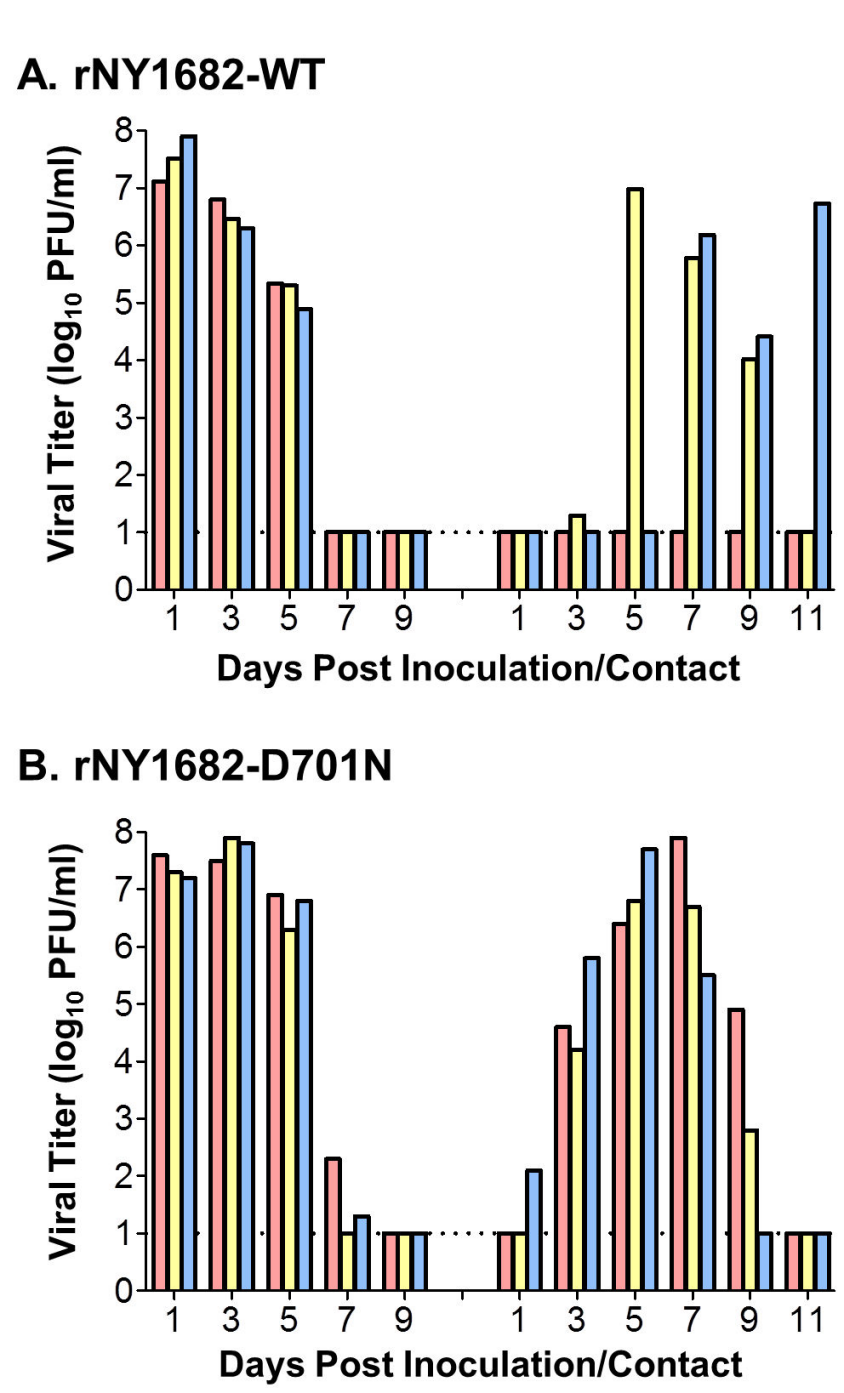
Respiratory droplet transmissibility of rNY1682-WT and rNY1682-D701N viruses in ferrets. Three ferrets were inoculated with 10^6^ PFU of (**A**) rNY1682-WT or (**B**) rNY1682-D701N virus. All ferrets were housed individually in specialized cages that permit exchange of respiratory droplets, but prevent direct or indirect contact between inoculated-contact animal pairs. A naïve ferret was placed in an adjacent cage to an inoculated ferret 1 day post-inoculation (0 day post-contact). Viral replication was determined by titration of nasal washes collected on alternate days from inoculated (left bars) and contact (right bars) ferrets. Values for individual ferrets are shown and the dotted lines at 1 log_10_ indicate the lower limit of detection of infectious virus.

We used the well-established ferret respiratory droplet transmission model to evaluate the effects of PB2-D701N on the transmissibility of H1N1pdm virus by housing ferrets in adjacent cages, each with a perforated side wall that prevented direct contact but allowed spread of virus through the air [26,33]. Three ferrets were inoculated intranasally with 10^6^ PFU of the rNY1682-WT or rNY1682-D701N virus, and 24 h later three naïve ferrets were each placed in a transmission cage adjacent to an inoculated ferret. Ferrets inoculated with rNY1682-WT and rNY1682-D701N viruses exhibited similar clinical signs including transient weight loss and fever before returning to baseline levels (data not shown). However, mean nasal wash virus titers from the rNY1682-D701N inoculated ferrets were significantly higher than that from the rNY1682-WT inoculated ferrets on day 3 and day 5 post inoculation (P<0.001, Figure 6A and 6B), consistent with the replication advantage of the rNY1682-D701N virus has in cells (human primary ATI-like cells, Calu-3 cells, and mouse CMT-93) and mice.

The ability of the viruses to undergo respiratory droplet transmission among ferrets was assessed by measuring virus titers in nasal washes from contact animals, collected at 1, 3, 5, 7, 9, and 11 days post-contact (dpc). The rNY1682-WT virus transmitted to two out of three contact ferrets (Figure 6A), demonstrating the same transmission efficiency as other H1N1pdm viruses previously evaluated in the same ferret transmission model [33–35]. This result was confirmed by the hemagglutation inhibition (HI) assay of the sera collected from these three ferrets, which showed that the two positive ferrets had HI antibody titers of 5280 and 2560 and the negative ferret had no detectable HI antibody (HI<10, at 18 dpc). In contrast, the rNY1682-D701N virus transmitted efficiently to all of the three contact ferrets (Figure 6B) and all of them were seroconverted (data now shown). Notably, in contrast to the largely delayed transmission of the rNY1682-WT virus, the rNY1682-D701N virus transmitted to contact ferrets as early as 1 dpc and all the ferrets showed relatively high virus titers by 3 dpc (Figure 6B).

Finally, to confirm our findings, an additional independent ferret transmission study was conducted with the same experimental design. In that study, we again found that the rNY1682-D701N virus transmitted to all three of the contact ferrets and with similar kinetics of transmission as was observed in the first study (in total 6/6 for PB2-701N on day 3 verses 4/6 for PB2-701D on day 7, Figure 6B and data not shown).

## Discussion

Asparagine (N) at residue 701 improves the binding of PB2 to mammalian importin-a isoforms [36–38], and for H5N1 and H7N7 avian influenza viruses it is associated with increased viral replication in mammalian cell lines, enhanced virulence in the mouse model, and more efficient transmission in the guinea pig model [12,16,28,39]. The data presented in this study demonstrate that the PB2-D701N substitution also increased the H1N1pdm virus replication in primary human cells, pathogenicity in the mouse model, and transmission in the ferret model. Notably, this is the first demonstration that PB2-D701N substitution enhances the transmissibility of influenza A viruses in the ferret transmission model.

Two other studies that examined the PB2-D701N and other PB2 mutations in the context of rA/Netherlands/602/2009 and rA/California/04/2009 H1N1pdm viruses concluded that both the PB2-E627K and PB2-D701N mutations had little impact on pathogenicity and transmissibility [6,7]. However, the supplemental data published by Yamada et al., showed the PB2-D701N substitution in rA/California/04/2009 H1N1pdm enhanced morbidity in BALB/cJ mice, which is consistent with our findings. However, the tissue titers of the rA/California/04/2009 PB2-701N virus were comparable with those of the PB2-701D virus at 3 dpi and 6 dpi, and the authors did not remark that PB2-D701N actually showed enhanced virulence as their supplemental data suggested [6]. One limitation with the study by Yamada et al. [6], is that tissue titers were not determined at early time points (e.g., 12, 24, and 48 hpi.), when higher levels of replication in the lungs are more pronounced (Figure 4), and thus differences from the wild type virus early in the course of infection would not have been evident. The data from Herfst et al.*,* suggested that the PB2-D701N substitution didn’t increase the transmission efficiency of the rA/Netherlands/602/2009 virus in their ferret transmission model [7]. The inconstant results between that study and the data presented in this study may result from differences in the virus strains used (four residues are different between the NY/1682 and Netherlands/602 strains), or the differences in the experimental design for the ferret transmission experiments. It appears that the transmission model for the wild type H1N1pdm (A/Netherlands/602/2009) used by Herfst et al.[7], is less stringent (4 of 4 contact ferrets are infected) than the transmission model used for this study, which consistently demonstrates that only two out of three contact ferrets show transmission with several other wild type H1N1pdm strains [33–35]. This more stringent transmission model allows one to detect the increased transmission rate upon introduction of the PB2-D701N substitution into the H1N1pdm virus and the data also clearly shows increased transmission kinetics (Figure 6). The faster transmission kinetics of the PB2-701N substitution may be related to the fact that this substitution enhances RdRp activity at cooler temperatures such as those found in the upper respiratory tract (Figure 1 D).

Collectively, using more detailed experimentation including using various cell lines at various temperatures, primary human lung cells, a well-designed mouse model, and a stringent ferret transmission model, we showed that the PB2-701N substitution enhanced the replication, pathogenesis, and transmission of an H1N1pdm virus in mice, ferrets, and primary cultured human alveolar epithelial cells. Therefore, we speculate that H1N1pdm viruses containing a PB2-D701N substitution may be more virulent and/or transmissible in humans than the viruses that circulated in the early years of the pandemic. Surprisingly, of the approximate 4,000 H1N1pdm PB2 sequences available in the major influenza virus databases (Influenza Research Database [40], NIH/NCBI Influenza Virus Resource [41], EpiFlu Database [42]), there is only one H1N1pdm virus (A/Wisconsin/51/2009) containing the PB2-D701N mutation (GISAID EpiFlu, accession#EPI273622, accessed 08-15-2012). The rarity of PB2 sequences with D701N mutation indicates that so far the mutation has not conferred better fitness to the H1N1pdm virus for transmission in humans, which is plausible since fitness and evolution in the human population is a polygenic trait. However, considering the estimated large number of infections worldwide [43], additional viruses with the PB2-D701N mutation may exist in the population. The higher polymerase activity and virus replication observed in human cells, and lower IFN-λ induction may give the virus an advantage by increasing mRNA transcription and genome replication. PB2 has been shown to inhibit interferon-β expression through its N-terminal region [36,44], and the mechanism underlying this IFN-λ suppression effect caused by a mutation in the C-terminal region is intriguing and warrants further investigation. With increased rounds of replication, additional mutations are more likely to occur, which may lead to emergence of other known or unknown virulence determinants [45]. Those virulence factors alone, or in combination with the PB2-D701N mutation, may increase the replication and pathogenicity of H1N1pdm viruses.

In summary, our findings support a role for the asparagine residue at PB2-701 in contributing to the virulence of influenza A viruses and show that PB2-D701N in the H1N1pdm virus increases its viral RNA polymerase activity in human and other mammalian cells, confers more efficient replication in cell lines and in primary human alveolar epithelial cells, and results in increased pathogenicity in mice and increased transmissibility in ferrets. The true threat of the PB2-D701N substitution to humans is unknown; however, if H1N1pdm viruses containing PB2-701N do emerge as this unique virus continues to evolve in human populations, it may pave the way to a more severe disease burden [39].
